# Surveillance of land molluscs infected by *Angiostrongylus cantonensis* (Nematoda) reveals risk areas for zoonotic eosinophilic meningitis in the State of Rio de Janeiro, Brazil

**DOI:** 10.1590/0074-02760240011

**Published:** 2025-02-03

**Authors:** Paulo Sergio Rodrigues, Suzete Rodrigues Gomes, Jucicleide Ramos-de-Souza, Monica Ammon Fernandez, Arnaldo Maldonado-Junior, Silvana Carvalho Thiengo

**Affiliations:** 1 Fundação Oswaldo Cruz-Fiocruz, Instituto Oswaldo Cruz, Laboratório de Malacologia, Rio de Janeiro, RJ, Brasil; 2 Fundação Oswaldo Cruz-Fiocruz, Instituto Oswaldo Cruz, Laboratório de Biologia e Parasitologia de Mamíferos Silvestres Reservatórios, Rio de Janeiro, RJ, Brasil

**Keywords:** Achatina fulica, Angiostrongylidae, zoonosis, nematode, epidemiological risk

## Abstract

**BACKGROUND:**

The nematode *Angiostrongylus cantonensis*, which is endemic to Southeast Asia and adjacent Pacific Islands, has already been recorded in more than 30 countries, including Brazil and other South American nations. It is one of the principal etiological agents of the zoonosis Eosinophilic Meningitis (EoM), which has a number of different species of terrestrial gastropods that act as its intermediate hosts.

**OBJECTIVE:**

The present study investigated the occurrence of the larvae of this nematode in specimens of terrestrial molluscs collected in half of the municipalities of the Brazilian State of Rio de Janeiro.

**METHODS:**

The study is based on the surveillance of this nematode in the Brazilian State of Rio de Janeiro, where terrestrial snails and slugs were collected in more than half of the state’s municipalities (46 in all), and examined for parasitological infections. The nematode larvae retrieved from these specimens were identified based on their morphology and cytochrome oxidase I (COI) mitochondrial DNA sequences.

**FINDINGS:**

Angiostrongylid larvae were found in 230 (8.8%) of the 2,600 terrestrial molluscs examined, collected from 26 municipalities. Overall, 14 terrestrial gastropod species were identified, including both native and exotic taxa, and six were found to be infected naturally by *A. cantonensis*. The natural infection rates by *Angiostrongylus* in the different terrestrial molluscs species were 12.5% in *Angustipes erinaceus*, 9.7% in *Achatina fulica*, 6.8% in *Bradybaena similaris*, 6.3% in *Sarasinula linguaeformis*, 3.9% in *Leptinaria unilamellata*, and 4.6% in *Subulina octona*. *A. fulica* was the most frequent and extensively distributed species, with infected snails being found in 22 municipalities.

**MAIN CONCLUSIONS:**

The data from this first comprehensive survey of *A. cantonensis* in Rio de Janeiro highlights the potential epidemiological risk of human infection in this state. Mapping the spread of infected molluscs will also provide essential information for the evaluation of the risk of human infection, and should help local health authorities to provide a faster and more accurate diagnosis whenever neuroangiostrongyliasis is suspected.

*Angiostrongylus cantonensis* (Chen, 1935) is a nematode parasite endemic to Southeast Asia and the adjacent Pacific Islands, and is one of the principal etiological agents of the zoonosis Eosinophilic Meningitis (EoM).^1^ ^,^ ^2^ ^,^ ^3^ ^,^ ^4^ Over the past three decades, this zoonosis has spread to a number of other countries around the world, with cases reported in humans or infected snails and rodents in Egypt, Madagascar, the United States, various Caribbean islands, Spain, Ecuador, Colombia, and Brazil.^5^ ^-^ ^15^

The biological cycle of *A. cantonensis* is complex, with the adult stage of the helminth being found in the arterial pulmonary system of rodents. In urban areas, the principal definitive hosts include *Rattus norvegicus* (Berkenhout, 1769) and *Rattus rattus* (Linnaeus, 1758), while the intermediate hosts include a number of different species of terrestrial snails, and the paratenic hosts are flatworms, crustaceans and fish, for example.^11^ ^,^ ^16^ The infection of humans by *A. cantonensis* is considered to be accidental and occurs mainly through the ingestion of snails and slugs parasitised with infective third stage (L_3_) larvae. However, the ingestion and the consumption of raw or poorly-cooked paratenic hosts, fruits or vegetables contaminated with larvae, is also a potential route of infection in humans.^10^ ^,^ ^17^

Up to now, approximately 40 cases of EoM have been confirmed in Brazil, based on records published in scientific journals, with more than 100 suspected cases in eight states ― Amapá, Espírito Santo, Pará, Paraná, Pernambuco, Rio de Janeiro, Rio Grande do Sul, and São Paulo.^3^ ^,^ ^9^ ^,^ ^14^ ^,^ ^15^ ^,^ ^18^ ^,^ ^19^ Eosinophilic meningitis is considered to be an emerging zoonosis in Brazil^14^ and a number of studies have associated the dissemination of this zoonosis with the spread of the exotic giant African snail, *Achatina fulica* Bowdich, 1822, an invasive species that has now spread to all 26 Brazilian states, as well as the Federal District.^4^ ^,^ ^20^ ^,^ ^21^

Given the wide distribution of this invasive snail and the risk of infection by *A. cantonensis* for human, wild, and domestic animals, the present study investigated the occurrence of the larvae of this nematode in specimens of terrestrial molluscs collected in half of the municipalities of the Brazilian State of Rio de Janeiro. The study used parasitological and genetic analyses to identify the focal sites of the transmission of *A. cantonensis*. The objective of this study was to identify these areas and to provide guidelines for the surveillance of the local populations of these animals and the prevention of the spread of EoM in the study area.

## MATERIALS AND METHODS

**Fig. 1:**
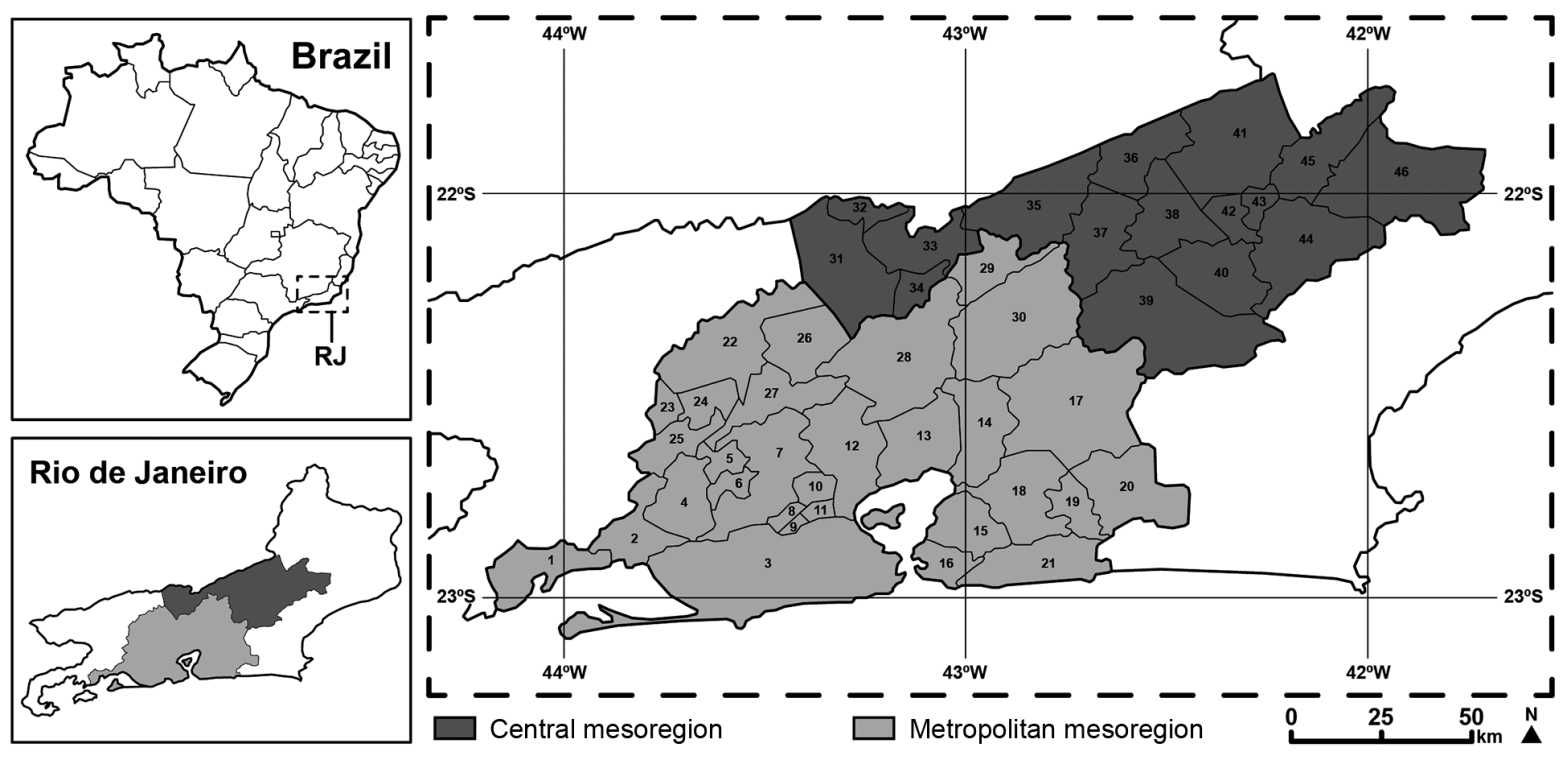
the 46 municipalities surveyed in the present study in the Metropolitan and Central (Centro Fluminense) mesoregions of the State of Rio de Janeiro, Brazil. 1-Mangaratiba, 2-Itaguaí, 3-Rio de Janeiro, 4-Seropédica, 5-Japeri, 6-Queimados, 7-Nova Iguaçu, 8-Mesquita, 9-Nilópolis, 10-Belford Roxo, 11-São João de Meriti, 12-Duque de Caxias, 13-Magé, 14-Guapimirim, 15-São Gonçalo, 16-Niterói, 17-Cachoeiras de Macacu, 18-Itaboraí, 19-Tinguá, 20-Rio Bonito, 21-Maricá, 22-Vassouras, 23-Mendes, 24-Engenheiro Paulo de Frontin, 25-Paracambi, 26-Paty do Alferes, 27-Miguel Pereira, 28-Petrópolis, 29-São José do Vale do Rio Preto, 30-Teresópolis, 31-Paraíba do Sul, 32-Comendador Levy Gasparian, 33-Três Rios, 34-Areal, 35-Sapucaia, 36-Carmo, 37-Sumidouro, 38-Duas Barras, 39-Nova Friburgo, 40-Bom Jardim, 41-Cantagalo, 42-Cordeiro, 43-Macuco, 44-Trajano de Moraes, 45-São Sebastião do Alto, 46-Santa Maria Madalena.

The Brazilian State of Rio de Janeiro has a total of 92 municipalities, which are divided into six mesoregions (Metropolitana do Rio de Janeiro, Centro Fluminense, Baixadas Litorâneas, Noroeste Fluminense, Norte Fluminense, and Sul Fluminense), as defined by the Brazilian Institute for Geography and Statistics (IBGE/2010). Two of these mesoregions, the Metropolitan and the Central (Centro Fluminense), together contain exactly half (46) of the state’s municipalities (Fig. 1), which are subdivided into 177 administrative districts. The Metropolitan mesoregion has more than 12,500,000 inhabitants (IBGE/2010) and is the second largest metropolitan area in Brazil (Censo/2010), with a total of 30 municipalities, many of which have a thriving tourism industry, a feature that also contributed to the selection of the study area.

The molluscs were collected manually from natural environments, such as vacant lots, parks, public squares, and peripheral areas with natural vegetation, in all of the 177 administrative districts that make up the study area, between February 2015 and March 2019. Each collection site was georeferenced, and the molluscs were taken to the National Reference Laboratory for Schistosomiasis and Malacology of the Instituto Oswaldo Cruz at the Fundação Oswaldo Cruz in Rio de Janeiro. In the laboratory, the specimens were maintained in captivity for one week for the identification of the species, based on their conchology and morphology, and then up to a month for the parasitological examination and the extraction of the nematode larvae by artificial digestion,^22^ with each specimen being processed individually. The larvae were separated under an optical microscope and identified initially to superfamily, based on their external morphology, with the specific objective of identifying the nematodes of the superfamily Metastrongyloidea.

In the case of the specimens identified as angiostrongylids, two subsamples were separated for morphological and molecular analyses. For the morphological procedure, 30 larvae per sample were fixed in heated AFA solution (2% glacial acetic acid, 3% formaldehyde, and 95% ethanol), to preserve their morphological structures, and stored in Eppendorf tubes. These specimens were clarified in lactophenol, mounted on slides, and examined under a bright field optical microscope for morphometry and the preparation of diagrams using a camera Lucida. For the molecular analysis, 10 larvae per sample were stored at -18ºC in phosphate-buffered saline (PBS) (1x) for the subsequent sequencing of the mitochondrial cytochrome oxidase I gene (COI), which has been used successfully as a DNA barcode for the diagnosis of species in a wide range of animal groups.^23^ The total number of nematodes per mollusc specimen was not estimated.

For the genetic analyses, the DNA was extracted by thermal shock with liquid nitrogen for 1 min and transferred immediately to a hot plate at 95ºC for 20 min, repeated three times. A 5 µL aliquot of this extract was reserved for amplification by polymerase chain reaction (PCR), using the protocol described by Prosser et al.:^23^ 1 x 94ºC for 5 min, 35 x (94ºC for 40 s, then 45ºC for 40 s, and 72ºC for 1 min), followed by 1 x 72ºC for 1 min, and then 1 x 17ºC for 3 min. The amplicon was then purified using the Illustra GFX kit for the purification of PCR DNA and gel banding (GE Healthcare, Little Chalfont, United Kingdom), following the manufacturer’s protocol, and then sequenced using the Sanger method in an ABI 3730xl automatic sequencer (Applied Biosystems) with 96 capillaries at the Oswaldo Cruz Institute Sequencing Platform in Rio de Janeiro.

To identify the species, the sequences obtained in the present study were compared with angiostrongylid sequences deposited in GenBank (www.ncbi.nlm.nih.gov/genbank/) using the Basic Local Alignment Search Tool (BLAST), available on the site of the National Centre for Biotechnology (NCBI). The BLAST search identified 22 *A*. *cantonensis* sequences of similar size, which were added to the matrix for the phylogenetic study. Two sequences representing other species of the genus *Angiostrongylus* were obtained from GenBank, and were added to the database as the outgroup.

The sequences were edited in Seqman 7.0 and aligned using Muscle,^24^ which was run in MEGA 11.0.13.^25^ The resulting sequence was trimmed to eliminate poorly-aligned extremities, and converted to the Nexus format using Mesquite 3.51.^26^ The Bayesian Inference (BI) analyses were run in MrBayes 3.2.7,^27^ with command blocks of the GTR + I nucleotide substitution model, which were calculated in MrModelTest and added to the matrix using Mesquite 3.7.^26^ The MrBayes analysis was run on the CIPRES Science Gateway V. 3.3.^28^ The BI node supports were calculated every 100 generations after the exclusion of a burn-in of 25%. To evaluate the quality of the sample, the Tracer 1.7.2 Software^29^ was used to calculate the effective sample size (ESS) of the parameters, with an ESS of over 200 being considered to be adequate for analysis.

## RESULTS

**Fig. 2:**
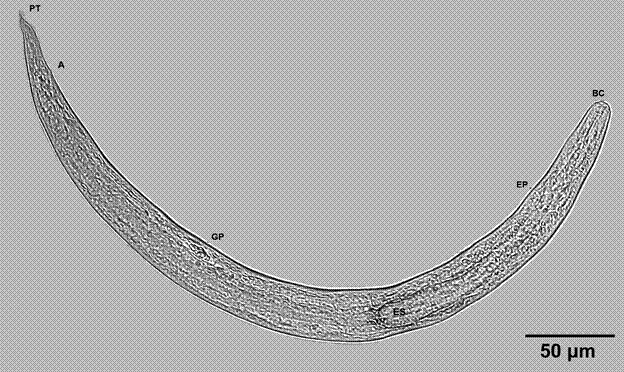
*Angiostrongylus cantonensis* larvae recovered from a terrestrial snail collected during the present study in Rio de Janeiro. PT: pointed tail; A: anus; GP: genital primordium; ES: oesophagus; BC: buccal cavity; EP: excretory pore.

Overall, 14 species of terrestrial molluscs, including both native and exotic taxa, were collected during the present study, with a total of 2600 specimens being available for parasitological analysis. Angiostrongylid larvae were observed in 230 of these specimens (8.8% of the total), while a further 1233 specimens (47.4%) were parasitised by nematodes of taxa other than the superfamily Metastrongyloidea. The diagnostic traits of the angiostrongylid larvae are the presence of a filariform oesophagus, pointed tail with bevelling, and a genital primordium (Fig. 2). The morphological structures of the larvae analysed here presented the diagnosis traits proposed by Ash^30^ and Thiengo et al.,^3^ which confirmed that they were angiostrongylids (Table I).

**TABLE I:** Morphometric parameters of the *Angiostrongylus* larvae collected from the Metropolitan and Central (Centro Fluminense) mesoregions of the Brazilian State of Rio de Janeiro, compared with the data of Ash^30^ and Thiengo et al.^3^ Values in micrometres

Parameter	Present study (n = 20)	Ash^30^	Thiengo et al.^3^
Total length	434 (420-489)	474 (425-524)	460 (460-544)
Width	24 (23-26)	26 (23-34)	25 (18-32)
Oesophagus	120 (101-125)	181 (167-194)	168 (129-189)
Excretory mouth-excretory pore	84 (79-92)	93 (78-105)	158 (80-200)
Genital primordium-end tail	121 (111-126)	174 (152-191)	41 (22-66)
Termination of tail	Pointed	Fine point	Pointed tip

The molecular analysis confirmed the occurrence of *A. cantonensis* in 26 (56.5%) of the 46 study municipalities (Table II). This nematode was found parasitising six species: the snails *A. fulica*, *Subulina octona* (Brugüière, 1789), *Bradybaena similaris* (Férussac, 1821) and *Leptinaria unilamellata* (d’Orbigny, 1835), and the slugs *Sarasinula linguaeformis* (Semper, 1885) and *Angustipes erinaceus* (Colosi, 1922). We also collected the snails *Bulimulus tenuissimus* (Férussac, 1832), *Drymaeus* sp., *Ovachlamys fulgens* (Gude, 1900), *Megalobulimus ovatus* (Müller, 1774), *Streptaxis contusus* (Férussac, 1821), *Tamayoa banghaasi* (Thiele, 1927), *Succinea* sp., *Beckianum beckianum* (Pfeiffer, 1846), but they were represented by shells or only few specimens that were not parasitologically examined. 

**TABLE II:** Terrestrial molluscs infected by *Angiostrongylus cantonensis* in the Metropolitan and Central (Centro Fluminense) mesoregions of the Brazilian State of Rio de Janeiro

Municipality	Mesoregion	Year	Terrestrial molluscs infected by *A. cantonensis*
Cachoeira de Macacu	Metropolitan	2015	*Achatina fulica*
Duque de Caxias	Metropolitan	2017	*Leptinaria unilamellata*
Itaboraí	Metropolitan	2015	*Achatina fulica*
Itaguaí	Metropolitan	2015	*Achatina fulica*
		2015	*Sarasinula linguaeformis*
Japeri	Metropolitan	2015	*Achatina fulica*
Magé	Metropolitan	2017	*Achatina fulica*
Mangaratiba	Metropolitan	2017	*Achatina fulica*
Maricá	Metropolitan	2016	*Achatina fulica*
Mendes	Metropolitan	2016	*Achatina fulica*
Mesquita	Metropolitan	2015	*Bradybaena similaris*
Miguel Pereira	Metropolitan	2018	*Achatina fulica*
Nilópolis	Metropolitan	2015	*Achatina fulica*
Niterói	Metropolitan	2015	*Achatina fulica*
		2017	*Sarasinula linguaeformis*
Nova Iguaçu	Metropolitan	2016	*Achatina fulica*
Paracambi	Metropolitan	2015	*Achatina fulica*
Queimados	Metropolitan	2016	*Subulina octona*
Rio Bonito	Metropolitan	2015	*Achatina fulica*
Rio de Janeiro	Metropolitan	2017	*Sarasinula linguaeformis*
		2019	*Achatina fulica*
São Gonçalo	Metropolitan	2015	*Achatina fulica*
Seropédica	Metropolitan	2015	*Achatina fulica*
Tanguá	Metropolitan	2015	*Achatina fulica*
		2015	*Leptinaria unilamellata*
Comendador Levy Gasparian	Central	2018	*Achatina fulica*
Santa Maria Madalena	Central	2017	*Achatina fulica*
Sapucaia	Central	2018	*Achatina fulica*
		2018	*Bradybaena similaris*
		2018	*Angustipes erinaceus*
Sumidouro	Central	2018	*Angustipes erinaceus*
Três Rios	Central	2018	*Achatina fulica*

**Fig. 3:**
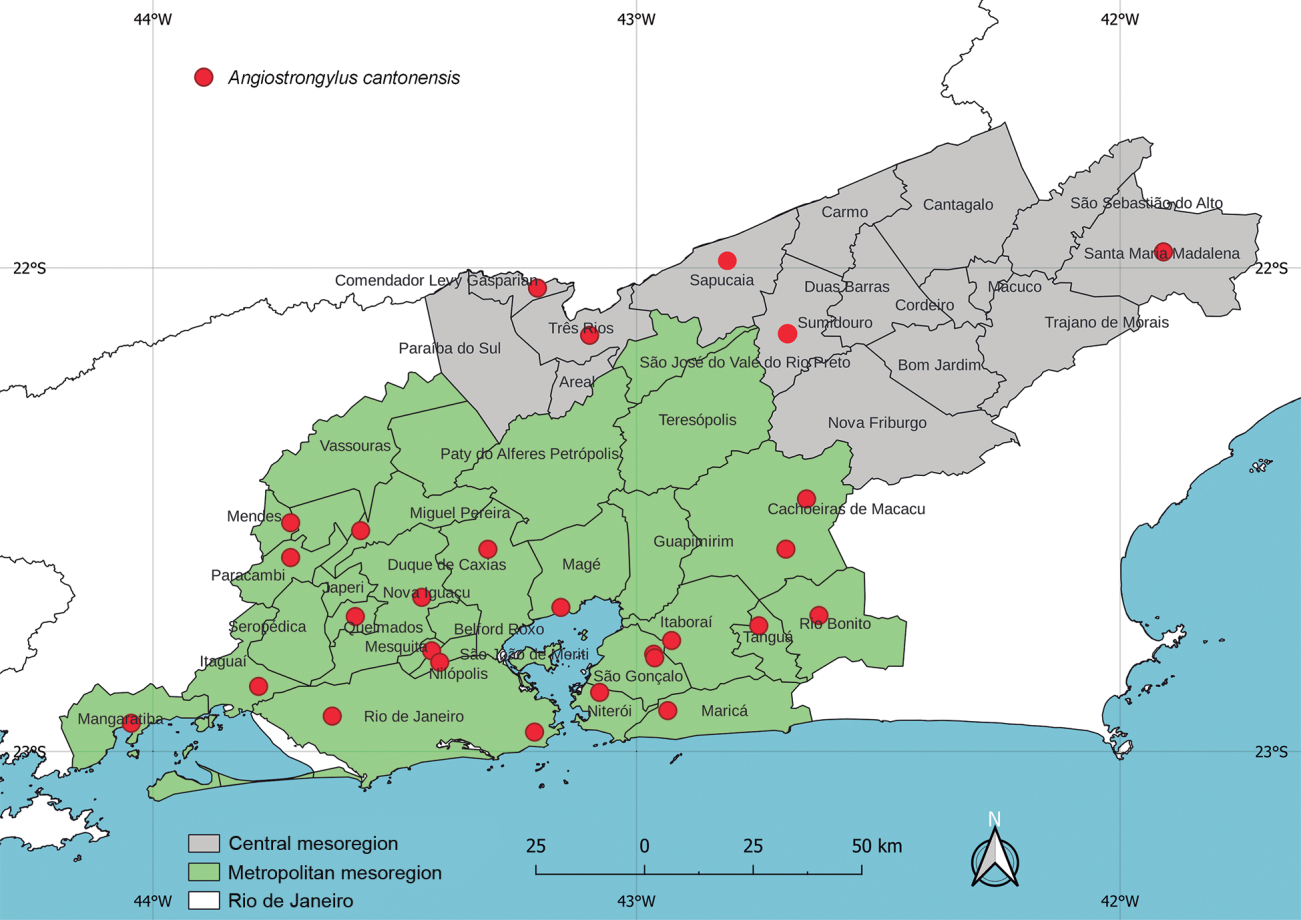
municipalities of the Metropolitan and Central (Centro Fluminense) mesoregions of Rio de Janeiro State which larvae of *Angiostrongylus cantonensis* were identified in the gastropod specimens (red dots).

The exotic invasive snail *A. fulica* was found infected by *A. cantonensis* in 22 municipalities, in samples collected in all four years of the study period. It is important to note that *A. fulica* was present in 41 of the 46 municipalities surveyed. Considering the study area, the natural infection rates by *Angiostrongylus* in the different gastropod species were 12.5% in *A. erinaceus*, 9.7% in *A. fulica*, 6.8% in *B. similaris*, 6.3% in *S. linguaeformis*, 3.9% in *L. unilamellata*, and 4.6% in *S. octona*. Overall, *A. cantonensis* was found in snails and slugs collected from natural biotopes in more than half (56.5%) of the municipalities surveyed in the present study. There was a clear predominance of infection in the Metropolitan mesoregion of the state (Fig. 3).

*Angiostrongylus cantonensis* was found infecting terrestrial molluscs collected from 21 of 30 of the municipalities (70%) of the Metropolitan mesoregion, but only five of the 16 municipalities (31%) of the Central mesoregion. While a much larger sample of molluscs (n = 2167) was collected in the Metropolitan mesoregion, given its larger number of municipalities, infection rates, which are a proportional measure, were also higher than those recorded in the Central mesoregion (Table III). A similar difference was also found in the number of terrestrial molluscs species, with five species being parasitised by *A. cantonensis* in the Metropolitan mesoregion, but only three in the Central mesoregion. The invasive *A. fulica* was the most frequent and widely distributed snail overall, and the species with the largest number of specimens parasitised by *A. cantonensis* in both mesoregions.

**TABLE III:** Number of terrestrial mollusc specimens collected in the Metropolitan and Central (Centro Fluminense) mesoregions of the Brazilian State of Rio de Janeiro, showing the species found to be infected with *Angiostrongylus cantonensis* and other nematodes results of the parasitological examination

	Specimens analysed (% of the total) in the:				Specimens positive (%) for *Angiostrongylus* in the:					Specimens positive (%) for other nematodes in the:	
Species	Metropolitan mesoregion		Central mesoregion		Metropolitan mesoregion		Central mesoregion			Metropolitan mesoregion	Central mesoregion
*Achatina fulica*	1715 (79.1)		353 (81.5)		185 (86.9)		15 (88.2)			904 (89.2)	192 (87.7)
*Bradybaena similaris*	116 (5.4)		46 (10.6)		10 (4.7)		1 (5.9)			60 (5.9)	18 (8.2)
*Sarasinula linguaeformis*	114 (5.3)		13 (3.0)		8 (3.8)		0			28 (2.8)	7 (3.2)
*Angustipes erinaceus*	1 (0.0)		7 (1.6)		0		1 (5.9)			0	2 (0.9)
*Subulina octona*	130 (6.0)		0		6 (2.8)		0			13 (1.3)	0
*Leptinaria unilamellata*	91 (4.2)		14 (3.2)		4 (1.9)		0			9 (0.9)	0
	2167		433		213		17			1014	219

	Specimens analysed in the:		Specimens positive for *Angiostrongylus* in the:		Specimens positive for other nematodes in the:		Both mesoregions positive (%)				
Molluscs	MT	CF	MT	CF	MT	CF	MT	CF	Both		
*Achatina fulica*	1715	353	185	15	904	192	10,8	4,2	9,7		
*Bradybaena similaris*	116	46	10	1	60	18	8,6	2,2	6,8		
*Sarasinula linguaeformis*	114	13	8	0	28	7	7,0	-	6,3		
*Angustipes erinaceus*	1	7	0	1	0	2	0,0	14,3	12,5		
*Subulina octona*	130	0	6	0	13	0	4,6	-	4,6		
*Leptinaria unilamellata*	91	14	4	0	9	0	4,4	-	3,8		
	2167	433	213	17							

CF: Centro Fluminense mesoregion; MT: Metropolitan mesoregion.

The molecular analysis of the larvae obtained from the six gastropod species produced COI sequences with a mean length of 600 base pairs (OR536625-OR536637, OR804433-OR804448). These sequences were highly similar to the *A. cantonensis* sequences available in the GenBank. Ten COI sequences were selected as reference sequences for the phylogenetic analysis, based on their size and quality (Table IV).

**TABLE IV:** Nematode sequences included in the phylogenetic analysis, showing the species, source country, host, and GenBank accession numbers

Family	Species	Country	Host	GenBank accession number	Reference on GenBank
Angiostrongylidae	*Angiostrongylus cantonensis*	Cambodia	*Pomacea* sp.	KY779735	Lv et al. 2018
		Cambodia	*Pomacea* sp.	KY779736	Lv et al. 2018
		Vietnam	*Pomacea* sp.	KY779737	Lv et al., 2018
		Vietnam	*Pomacea* sp.	KY779738	Lv et al. 2018
		United States	*Didelphis virginiana*	MF000735	Dalton et al. 2017
		United States	*Didelphis virginiana*	MF000736	Dalton et al. 2017
		Taiwan	*-*	AP017672	Unpublished
		Australia	*Rattus fuscipes*	MN814826	Valentyne et al. 2020
		Australia	*Rattus rattus*	MN814827	Valentyne et al. 2020
		Australia	*Rattus rattus*	MN814828	Valentyne et al. 2020
		French Polynesia	*Rattus exulans*	MK570632	Cervená et al. 2019
		Australia	*Rattus rattus*	MK570631	Cervená et al. 2019
		Spain	*Rattus rattus*	MK570629	Cervená et al. 2019
		United States	*Rattus exulans*	MK570630	Cervená et al. 2019
		Thailand	*Mus musculus*	KT947978	Yong et al. 2016
		China	*Rattus norvegicus*	GQ398121	Lv et al. 2012
		Brazil	*Achatina fulica*	MN994436	Barbosa et al. 2021
		Brazil	*Achatina fulica*	MN994437	Barbosa et al. 2021
		Brazil	*Achatina fulica*	MN994438	Barbosa et al. 2021
		Brazil	*Bulimulus tenuissimus*	MH547424	Ramos-de-Souza et al. 2018
		Brazil	*Cyclodontina fasciata*	MH511542	Ramos-de-Souza et al. 2018
		Brazil	*Achatina fulica*	MH511539	Ramos-de-Souza et al. 2018
		Brazil	*Leptinaria unilanellata*	-	Present study
		Brazil	*Sarasinula liguaeformis*	-	Present study
		Brazil	*Achatina fulica*	-	Present study
		Brazil	*Achatina fulica*	-	Present study
		Brazil	*Achatina fulica*	-	Present study
		Brazil	*Achatina fulica*	-	Present study
		Brazil	*Achatina fulica*	-	Present study
		Brazil	*Bradybaena similaris*	-	Present study
		Brazil	*Angustipes erinaceus*	-	Present study
		Brazil	*Subulina octona*	-	Present study
	*Angiostrongylus malaysiensis*	Malaysia	*Rattus rattus*	KT947979	Hoi-Sen Yong et al. 2016
	*Aelurostrongylus abstrusus*	Australia	*Felis silvestris catus*	NC019571	Jabbar et al. 2013

**Fig. 4:**
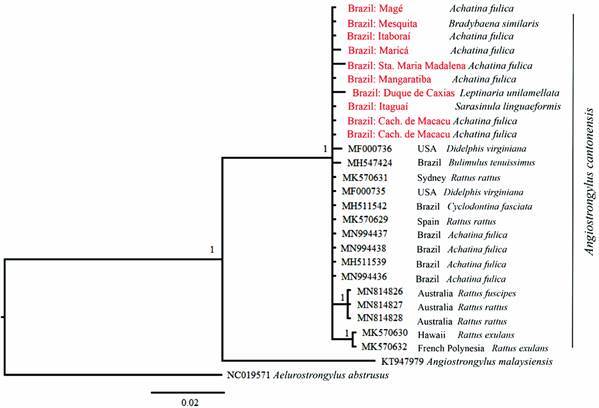
phylogenetic tree generated by Bayesian inference from the mitochondrial cytochrome oxidase I (COI) gene sequences obtained during the present study. The samples are identified by their GenBank accession codes, country (or municipality, in the case of the present study), the nematode species (*Angiostrongylus cantonensis*), country, and the host species.

The GTR + I nucleotide substitution model was selected by the Akaike Information Criterion (AIC), run in MrModelTest, as having the best adjustment for the COI matrix compiled here. The BI phylogenetic tree (Fig. 4) allocated all the samples to a single clade. As the ESS values were well above 200, the sampling was considered to be adequate.

## DISCUSSION

Natural infection by *A. cantonensis* was observed in six of the total of 14 species collected in the present study in Rio de Janeiro State, which reconfirms the lack of specificity of this nematode in relation to its intermediate hosts, as reported by Caldeira et al.,^9^ Thiengo et al.^4^ and Valente et al.^31^ A similar pattern has also been observed in endemic regions. After screening the principal Hawaiian islands to determine which gastropods act as hosts of *A. cantonensis*, Kim et al.^32^ recorded the presence of the parasite in 16 species (two native, 14 non-native) of a total of 37 species, with 70 of the 1271 specimens testing positive. In this study,^32^ the small gastropod *S. octona* had the highest mean concentration of parasites, while the veronicellid slug *Laevicaulis alte* had the highest individual parasite concentration. Kim et al.^32^ identified only seven positive specimens of *A. fulica* in a sample of 62 (11%), and added three gastropod families to the 33 already known to host *A. cantonensis*. Jaume-Ramis et al.^33^ recorded a similar scenario in Mallorca, Spain, where they collected and identified 398 gastropods belonging to 17 species (14 snails and three slugs), of which 11% tested positive for *A. cantonensis*. The gastropods were collected at sites ranging from tourist settlements to farmland, with the least abundant species being the less likely to be infected. *A. fulica* was not recorded in this study. Santana-Teles et al.^34^ and Prociv et al.^6^ also reported a lack of specificity in the *A. cantonensis* vertebrate hosts. All the gastropod species found infected with *A. cantonensis* in the present study, except *L. unilamellata*, had already been reported being infected with this nematode.

The larger numbers of infected molluscs found in the Metropolitan mesoregion highlight the risk of the occurrence of cases of EoM in the urban areas of these municipalities, which are densely populated, in general, and tend to have large concentrations of domestic animals. Two other *Angiostrongylus* species are known to both occur in Brazil and parasitise molluscs, *Angiostrongylus costaricensis* (Morera & Céspedes, 1971), a parasite of rodents which can infect humans accidentally, and causes abdominal angiostrongyliasis, and *Angiostrongylus vasorum* (Baillet, 1866), a cardio-pulmonary parasite of canids. While neither of these species was detected in the present study, their potential occurrence in the study area cannot be ruled out altogether, given that *Angiostrongylus* nematodes tend to be relatively unspecific in terms of their intermediate and definitive hosts.

Turck et al.^35^ reviewed the reports of *A. cantonensis* infections in both academic papers and the grey literature, and found that the number of human cases of infection had more than doubled worldwide over the past 10 years. Brazil is likely to have undergone a similar increase in infection levels, given that infected *A. fulica* have now been recorded in 14 of the country’s 26 states, and in its Federal District.^4^ ^,^ ^36^ ^,^ ^37^ ^,^ ^38^ ^,^ ^39^ ^,^ ^40^ Human cases of *A. cantonensis* infection have now been reported from southern, southeastern, northeastern, and northern Brazil.^9^ ^,^ ^14^ ^,^ ^15^ ^,^ ^20^

In addition, the very large population of *A. fulica* observed in the present study, together with the *A. cantonensis* infection rates, indicate that this snail may represent the principal intermediate host of this parasite in Rio de Janeiro, considering its ample distribution and dense populations, in particular in urban areas. The proximity of this snail to humans, especially in urban environments, where its coexistence with synanthropic rodents establishes an important link in the epidemiology of *A. cantonensis*, increases the risk of human infection.^36^ ^,^ ^37^ ^,^ ^38^ ^,^ ^39^ ^,^ ^40^ ^,^ ^41^

In comparison with the Central mesoregion, *A. fulica* was much more common in the Metropolitan mesoregion, which has a densely-populated urban centre, with many social and environmental problems, including the proliferation of low-income housing in areas that lack public sanitation or adequate garbage disposal. This favours the establishment of invasive species that act as urban pests, and are associated with the transmission of diseases to humans and domestic animals. The Central mesoregion has a much lower population density, however, with more rural areas.^42^

In a previous study in the Brazilian state of Rio de Janeiro, Oliveira et al.^37^ reported *A. fulica* and *B. similaris* being parasitised by *A. cantonensis*, with a prevalence of infection by *A. cantonensis* of more than 50% in *A. fulica* throughout the study area*.* In another study, Kim et al.^32^ concluded that the level of infection of *A. fulica* varies widely among the different Hawaiian Islands and that the dissemination of *A. cantonensis* is most likely determined by local habitats, rather than the presence of *A. fulica*. These authors related the observed differences in infection rates primarily to abiotic factors such as temperature and humidity, and secondarily to the distribution of infected rats, the rat species on the different islands and the variation in the level of interaction between the rats and the local gastropods.

The results of the present study highlight the epidemiological risk of transmission of EoM in the study area, based on the wide distribution of *A. cantonensis* and the high densities of its intermediate host molluscs. Knowledge of this epidemiological risk, combined with the existing sound diagnostic criteria,^43^ should enable the faster and more reliable detection of EoM by the local health services.

*In conclusions* - The present study showed that *A. cantonensis* is now widespread in both the Metropolitan and the Central mesoregions of the State of Rio de Janeiro, Brazil, and highlights the potential epidemiological risk of human infection in this state. The transmission of *A. cantonensis* is associated with six species of terrestrial molluscs, with *A. fulica* having an important role in the dissemination of this nematode, considering that it is the most frequent and widely-distributed snail, overall, in both mesoregions.

Understanding the distribution of infected intermediate hosts in the different municipalities will be extremely useful for epidemiological surveillance and investigations by public health services and should help local health authorities to provide a faster and more accurate diagnosis whenever neuroangiostrongyliasis is suspected.

## References

[1] Alicata JE (1991). The discovery of Angiostrongylus cantonensis as a cause of human eosinophilic meningitis. Parasitol Today.

[2] Graeff-Teixeira C, Da Silva ACA, Yoshimura K (2009). Update on eosinophilic meningoencephalitis and its clinical relevance. Clin Microbiol Rev.

[3] Thiengo SC, Maldonado A, Mota EM, Torres EJL, Caldeira R, Carvalho OS (2010). The giant African snail Achatina fulica as natural intermediate host of Angiostrongylus cantonensis in Pernambuco, northeast Brazil. Acta Trop.

[4] Thiengo SC, Ramos-de-Souza J, Silva GM, Fernandez MA, Silva EF, Sousa AKP (2022). Parasitism of terrestrial gastropods by medically-important nematodes in Brazil. Front Vet Sci.

[5] Kliks MM, Palumbo NE (1992). Eosinophilic meningitis beyond the Pacific Basin the global dispersal of a peridomestic zoonosis caused by Angiostrongylus cantonensis, the nematode lungworm of rats. Soc Sci Med.

[6] Prociv P, Spratt DM, Carlisle MS (2000). Neuro-angiostrongyliasis unresolved issues. Int J Parasitol.

[7] Lindo JF (2002). Enzootic Angiostrongylus cantonensis in rats and snails after an outbreak of human eosinophilic meningitis, Jamaica. Emerg Infect Dis.

[8] Raccurt CP, Blaise J, Durette-Desset MC (2003). Presence of Angiostrongylus cantonensis in Haiti. Trop Med Int Health.

[9] Caldeira RL, Mendonça CLGF, Goveia CO, Lenzi HL, Graeff-Teixeira C, Lima WS (2007). First record of molluscs naturally infected with Angiostrongylus cantonensis (Chen, 1935) (Nematoda Metastrongylidae) in Brazil. Mem Inst Oswaldo Cruz.

[10] Delgado-Serra S, Sola J, Negre N, Paredes-Esquivel C (2022). Angiostrongylus cantonensis nematode invasion pathway, Mallorca, Spain. Emerg Infect Dis.

[11] Maldonado A, Simões RO, Oliveira APM, Motta EM, Fernandez MA, Pereira ZM (2010). First report of Angiostrongylus cantonensis (Nematoda Metastrongylidae) in Achatina fulica (Mollusca: Gastropoda) from Southeast and South Brazil. Mem Inst Oswaldo Cruz.

[12] Carvalho OS, Scholte RGC, Mendonça CLF, Passos LKJ, Caldeira RL (2012). Angiostrongylus cantonensis (Nematode Metastrongyloidea) in molluscs from harbour areas in Brazil. Mem Inst Oswaldo Cruz.

[13] Simões R (2011). Endemic Angiostrongyliasis, Rio de Janeiro, Brazil. Emerg Infect Dis.

[14] Morassutti AL, Thiengo SC, Fernandez M, Sawanyawisuth K, Graeff-Teixeira C (2014). Eosinophilic meningitis caused by Angiostrongylus cantonensis an emergent disease in Brazil. Mem Inst Oswaldo Cruz.

[15] Barbosa TA, Thiengo SC, Fernandez MA, Graeff-Teixeira C, Morassutti AL, Mourão FRP (2020). Infection by Angiostrongylus cantonensis in both humans and the snail Achatina (Lissachatina) fulica in the city of Macapá, in the Amazon Region of Brazil. Mem Inst Oswaldo Cruz.

[16] Guerino LR, Pecora IL, Miranda MS, Aguiar-Silva C, Carvalho OS, Caldeira RL (2017). Prevalence and distribution of Angiostrongylus cantonensis (Nematoda, Angiostrongylidae) in Achatina fulica (Mollusca, Gastropoda) in Baixada Santista, São Paulo, Brazil. Rev Soc Bras Med Trop.

[17] Cowie RH (2013). Biology, systematics, life cycle, and distribution of Angiostrongylus cantonensis, the cause of rat lungworm disease. Hawaii J Med Public Health.

[18] Lima ARMC, Mesquita SD, Santos SS, Aquino ERP, Rosa LRS, Duarte FS (2009). Alicata disease neuroinfestation by Angiostrongylus cantonensis in Recife, Pernambuco, Brazil. Arq Neuro-Psiquiatr.

[19] Espírito-Santo MCC, Pinto PLS, Mota DJG, Gryschek RCB (2013). The first case of Angiostrongylus cantonensis eosinophilic meningitis diagnosed in the city of São Paulo, Brazil. Rev Inst Med Trop São Paulo.

[20] Ramos-de-Souza J, Gomes SR, Mattos AC, Sousa AKP, Silva EF, Maldonado-Junior A (2023). Achatina fulica infected by Angiostrongylus cantonensis in Manaus, Brazilian Amazon Region, and the risk of transmission of eosinophilic meningitis. J Trop Pathol.

[21] Arruda JO, Santos L (2022). First record of Achatina fulica Bowdich, 1822 (Mollusca, Achatinidae), for the State of Rio Grande do Sul, Brazil. Biotemas.

[22] Graeff-Teixeira C, Morera P (1995). Método de digestão de moluscos em ácido clorídrico para isolamento de larvas de metastrongilídeos. Biociências.

[23] Prosser SWJ, Velarde-Aguilar MG, León-Règagnon V, Hebert PDN (2013). Advancing nematode barcoding a primer cocktail for the cytochrome c oxidase subunit I gene from vertebrate parasitic nematodes. Mol Ecol Resour.

[24] Edgar RC (2004). MUSCLE a multiple sequence alignment method with reduced time and space complexity. BMC Bioinformatics.

[25] Tamura K, Stecher G, Kumar S (2021). MEGA11 Molecular Evolutionary Genetics Analysis Version 11. Mol Biol Evol.

[26] Maddison WP, Maddison DR (2019). Mesquite: a modular system for evolutionary analysis. version 3.61.

[27] Ronquist F, Teslenko M, van der Mark P.Ayres DL.Darling A.Höhna S (2012). MrBayes 3 2: efficient bayesian phylogenetic inference and model choice across a large model space. Syst Biol.

[28] Miller MA, Pfeiffer W, Schwartz T (2010). Creating the CIPRES Science Gateway for inference of large phylogenetic trees. IEEE.

[29] Rambaut A, Drummond AJ, Xie D, Baele G, Suchard MA (2018). Posterior summarization in Bayesian phylogenetics using tracer 1 7. Syst Biol.

[30] Ash LR (1970). Diagnostic morphology of the third-stage larvae of Angiostrongylus cantonensis, Angiostrongylus vasorum, Aelurostrongylus abstrusus, and Anafilaroides rostratus (Nematoda Metastrongyloidea). J Parasitol.

[31] Valente R, Robles MR, Diaz JI (2020). Gastropods as intermediate hosts of Angiostrongylus spp in the Americas: bioecological characteristics and geographical distribution. Mem Inst Oswaldo Cruz.

[32] Kim JR, Hayes KA, Yeung NW, Cowie RH (2014). Diverse gastropod hosts of Angiostrongylus cantonensis, the rat lungworm, globally and with a focus on the Hawaiian Islands. PLoS One.

[33] Jaume-Ramis S, Martínez-Ortí A, Delgado-Serra S, Bargues MD, Mas-Coma S, Foronda P (2023). Potential intermediate hosts of Angiostrongylus cantonensis in the European Mediterranean region (Mallorca, Spain). One Health.

[34] Teles HMS, Vaz JF, Fontes LR, Domingos MF (1997). Registro de Achatina fulica Bowdich, 1822 (Mollusca, Gastropoda) no Brasil caramujo hospedeiro intermediário da angiostrongilíase. Rev Saude Publica.

[35] Turck HC, Fox MT, Cowie RH (2022). Paratenic hosts of Angiostrongylus cantonensis and their relation to human neuroangiostrongyliasis globally. One Health.

[36] Thiengo SC, Fernandez MA (2010). Achatina fulica: um problema de saúde pública? O caramujo gigante africano Achatina fulica no Brasil.

[37] Oliveira APM, Gentile R, Maldonado A, Torres EJL, Thiengo SC (2015). Angiostrongylus cantonensis infection in molluscs in the municipality of São Gonçalo, a metropolitan area of Rio de Janeiro, Brazil role of the invasive species Achatina fulica in parasite transmission dynamics. Mem Inst Oswaldo Cruz.

[38] Ramos-de-Souza J, Thiengo SC, Fernandez MA, Gomes SR, Corrêa-Antônio J, Clímaco MC (2018). First records of molluscs naturally infected with Angiostrongylus cantonensis (Nematoda Metastrongyloidea) in Sergipe State, Northeastern Brazil, including new global records of natural intermediate hosts. Rev Inst Med Trop São Paulo.

[39] Almeida LR, Souza Joaquim J, Botelho LM, Vidigal THDA, Ecco R, Souza Trindade G (2023). Parasitism in Rattus rattus and sympatric Achatina fulica by Angiostrongylus cantonensis in an urban park in southeast Brazil. Parasitol Res.

[40] Graeff-Teixeira C (2007). Expansion of Achatina fulica in Brazil and potential increased risk for angiostrongyliasis. Trans R Soc Trop Med Hyg.

[41] Pilate VJ, Chicarino ED, Silva LC, Santos TV, Souza BA, Bessa ECA (2017). Biologia comportamental comparada entre moluscos terrestres nativos e exóticos. Rev Biol Neotrop.

[42] Souza RP (2019). O desenvolvimento rural no estado do Rio de Janeiro a partir de uma análise multidimensional. RESR.

[43] Graeff-Teixeira C, Sawanyawisuth K, Lv S, Sears W, Rodríguez ZG, Álvarez HH (2023). Neuroangiostrongyliasis updated provisional guidelines for diagnosis and case definitions. Pathogens.

